# The Psychological Impact of Restorative Justice Practices on Victims of Crimes—a Systematic Review

**DOI:** 10.1177/15248380221082085

**Published:** 2022-04-23

**Authors:** Ana M. Nascimento, Joana Andrade, Andreia de Castro Rodrigues

**Affiliations:** 1 56068 William James Center for Research, ISPA, Lisboa, Portugal; 2 Epsi-UM, Braga, Portugal

**Keywords:** systematic review, restorative justice, conference/mediation victim–offender, victims, psychological impacts

## Abstract

Background: Restorative justice emerges as a theoretical-practical approach to the criminal legal system, in which the reparation of damage of the victim is a central point. However, the growing empirical production referring to the effects of this approach on victims is sometimes shown to be weakened or dispersed, focusing mainly on their satisfaction. Objective: The present work intended to systematically evaluate the empirical production of the restorative justice field, to aggregate and examine information in the literature regarding the psychological impacts on victims who participated in restorative practices. Methods: A search was made using electronic databases to identify quantitative, qualitative, and mixed-method studies, published between January 2000 and December 2020 that reported psychological impacts on real victims of crimes, who participated in mediations/conferences victim–offender. Results: 35 studies were identified as focusing on the psychological impacts on victims resulting from restorative practices. These studies have shown effects on post-traumatic symptomatology, on the emotions and emotional needs resulted from victimization, as well as on the victims’ perceptions of their offenders. Conclusions: The present research showed that restorative justice practices have a positive psychological impact on victims, who are frequently forgotten in conventional justice, and that some of these impacts persist over time.

## Introduction

Restorative Justice (RJ) is an approach to criminal legal systems that emerges as an alternative to the so-called conventional justice (CJ) (UN, 2002). It aims to be a response to the lack of a holistic and humanizing view, felt by the parties involved in a crime, due to the absence of more inclusive strategies that meet the needs of victims and, consequently, minimize their psychological damage (Strang, 2002; Wemmers & Cyr, 2005J. A. Wemmers & Cyr, 2005; Zehr, 2005). Choi and Severson (2009) reported that many crime victims face insensitive treatments by traditional criminal justice systems, often feeling excluded from their own processes. The same authors also mention that, in addition, victims frequently do not receive any restitution and rarely hear genuine expressions of remorse by the offenders when the case is conducted through the procedures of CJ. According to Zehr (2005), the result of these gaps, that conduct to the dissatisfaction of victims, was the kick off for contemporary developments of restorative approaches in criminal justice systems and the encouragement by the United Nations Office on Drugs and Crime (2006) for the adoption of RJ among its State Members. The intention was to promote a more flexible, comprehensive, and humanistic perspective of the legal system, to facilitate the restoration/recovery processes of victims, offenders and communities.

Despite the inexistence of a single and consensual definition for RJ, a common idea of most conceptualizations is that RJ consists in as a theoretical-practical approach, which takes place within the criminal justice system and has the purpose to strive redress for the damage caused by crimes, assuming a more inclusive and participatory nature (Braithwaite, 2002). Thus, instead of seeing the criminal act as a violation of the law that leads to the punishment of an offender (Strang, 2002; UN, 2002), RJ considers the crime as an irregular action that caused harm to an individual or community, and therefore a criminal act is seen as a violation of a person and the relationships between people (victim–offender). This innovative conception of crime (i.e., as an interpersonal transgression), intends to promote accountability (in a significant way), as well as the offenders’ moral obligation to repair the damage caused by their actions and to seek the restoration of the affected relationship (Zehr, 2005). To fulfill this goal, RJ presupposes that victims, offenders, and the community (or their representatives) come together to talk about the incident and engage in a “restorative dialogue.” The most common form of these encounters is the victim–offender mediation or, according to some terminology, victim–offender conference (an extended form of mediation) (Johnstone & Van Ness, 2013), which were the first types of RJ meeting to be established and are, even today, the most used models in RJ programs (Umbreit, 2002) and the ones that reports higher satisfaction levels on its participants (McCold & Watchel, 2002).

In addition, Zehr (2015) also states that these RJ processes aim to balance the spheres of power among the participants, seeking victim empowerment, while providing sensitive support to the offender. This balance facilitates the process of communication and productive dialogue, avoiding on the one hand that both parties are in opposition, and on the other hand that one is more vulnerable than the other. Thus, RJ practices require a unique and rigorous framework, guided by a set of universal values, such as justice, solidarity and responsibility, respect for human dignity and truth (EFRJ, 2020).

Despite the popularity of RJ programs, research regarding its evaluation is still in an embryonic state, since the majority of the literature focuses on the analysis of stakeholder satisfaction, especially the offender perspective. In this sense, it is often suggested by researchers that participation in RJ programs has a beneficial impact on the level of satisfaction for victims and offenders (Bonta et al., 2002; Latimer et al., 2005; Rugge et al., 2006; Strang, 2002; Umbreit et al., 1994). Given the importance of the participant satisfaction with their restorative processes, some investigators have exclusively examined this indicator in determining the success of RJ programs (Dignan, 2005; Umbreit et al., 1994). However, since high levels of satisfaction do not necessarily indicate the effectiveness of a program such analysis seems to be reductive and insufficient (Zehr, 2005).

Besides, recent empirical production has shown several effects on victims, which go far beyond satisfaction and include positive psychological outcomes. From the restorative paradigm, the literature highlights the importance of voluntary participation by victims, not only for the opportunity to express their thoughts and emotions regarding what happened, but also for feeling heard, which leads to their validation (Strang et al., 2006; Umbreit, 2002). Moreover, RJ fosters a greater involvement of victims in their own processes and promotes the access to significant information about their victimization (“why me?”) (Strang, 2002). Thus, Zehr (2005) states that this way the conditions are created for victims to better integrate and re-signify their victimization experiences. Also, the narration of trauma, whether public or private, as occurs in RJ practices, relieves sadness, mitigates fears and anxieties, and promotes the repair of those who have suffered damage (Neimeyer et al., 2006). Consistent with these results, Lloyd and Borril (2019) reveal the significant effects of RJ practices improving post-traumatic stress symptomatology (PTSS) in victims, closely related to aspects such as avoidance, intrusive thoughts and hyperreactivity, which are based, respectively, on the victim levels of fear, distress, and anxiety.

Furthermore, other studies show that RJ practices give victims a greater sense of control (empowerment), as they can decide the degree of involvement they intend to have in the processes (Gustafson, 2005). According to victimology literature, victims’ empowerment is the basis to restore both the sense of security and self-confidence (Kellas & Manusov, 2003), which are needed to the transformative movement from victim status to survivor^1^ status (Brosi & Rolling, 2010; Leisenring, 2006; SAKI, 2021). This movement is based on the acceptance of losses and in the consequent emotional overcome (closure) of the situation (Beck et al., 2015; Hoff & Hoff, 2011). Thus, it is not surprising that the justice professionals involved (e.g., lawyers, mediators) also report that RJ approaches have remarkably more positive results for victims when compared to conventional interventions (Bonta et al., 2002; Latimer et al., 2005).

Most studies highlight, both the reduction of negative emotions and feelings such as anger, fear, anxiety, distress, and sadness (Kunst & Varejamp, 2014; Strang, 2002; Umbreit et al., 2004), and the development of more positive, empathic and humanized perceptions toward offenders (Braithwaite, 2002). However, the literature suggests that the psychological consequences of victimization are likely to vary depending on several aspects: the type of crime (i.e., whether low or high severity); the levels of damage experienced; and the individual characteristics of the victim and the offender (Shapland & Hall, 2007). Other previous studies advocated that the levels of satisfaction of victims in restorative processes would be related to the perceptions of justice (i.e., having their rights recognized and their victimization validated) and with the sense of their needs being met (Latimer et al., 2005; Rugge et al., 2006; Strang, 2002).

Despite quantitative analysis point to high levels of victim satisfaction with their restorative experiences, Choi et al. (2012), in their qualitative literature review, focused on cases of victim dissatisfaction in these processes, referring to the feeling that the offender remorse and apologies were not entirely sincere, thus jeopardizing the restorative outcome of the mediations. The same authors identified gaps between RJ theory and practice, pointing to the extreme importance of the proper training of mediators, who should be able to establish effective communications during meetings, avoid the danger of the victim becoming morally dominant or be revictimized during the restorative process, and consequently get an unsatisfactory psychological result.

Although positive assessments of victims’ levels of satisfaction with their restorative experiences predominate (Latimer et al., 2005; Strang, 2002), there are few studies that specifically examine the psychological impacts on participating victims of RJ, when compared to the so-called traditional types of legal proceedings. However, the fact that many studies report that victims show high levels of satisfaction with RJ does not necessarily allow to infer the existence of more positive psychological impacts. Thus, considering the possible psychological consequences of victimization processes (i.e., negative emotions, distress, PTSS, emotional needs), it becomes necessary to understand how restorative practices can affect relevant psychological outcomes on victims of crime (Lloyd & Borril, 2019). In addition, despite the global popularity of RJ and the growth of evidence regarding its psychological impacts, data are dispersed throughout the literature. That is, specific issues of psychological well-being are often confused or concealed in the literature with studies that focus on victim satisfaction levels (Angel, 2014). Moreover, the reviews recently founded in the literature are mainly qualitative (and non-systematic), or highlight exclusively specific outcomes (Choi et al., 2012; Lloyd & Borril, 2019). Thus, given the need to develop a systematic review of the literature, this work intends to condense the scattered information regarding the psychological impacts of RJ on crime victims, which complements and deepens the assessment of general satisfaction indices.

## Method

### Eligibility Criteria

Eligibility criteria for the inclusion of studies in this review were developed based on the standardized SPIDER strategy (Cooke et al., 2012). This strategy presupposes the definition of five key elements for the review, namely: (S) Sample, that should reflect the group of interest; (PI) Phenomenon of Interest, which refers to the type of experiences and interventions to be investigated; (D) Design, as the theoretical approach determines the type of research used (e.g., experimental, quasi-experimental, correlational, descriptive, among others); (E) Evaluation, which concerns the results of the studies; and (R) Research type which may be qualitative, quantitative, or mixed-method (Cooke et al., 2012).

Thereby, the present study focuses its analysis on victims of crimes, excluding studies in which the victims are not real (that is, studies whose sample consists of people invited to imagine themselves as having been victimized). Similar to previous studies, this work considered victims of murder crimes as the closest relative of the murdered victim.

The phenomenon of interest in this review referred to interventions based on restorative practices in the criminal sphere, this is victim–offender meetings (VOM), particularly mediations or conferences. These typologies of intervention have higher levels of structure and are the most used in RJ programs because, according to the literature, they are the kind of practices that shows more evidence of effects (i.e., satisfaction) on participants (McCold & Watchel, 2002).

In the present work, we considered for analysis all the results of relevant quantitative, qualitative or mixed-method empirical studies, capable of elucidating about psychological impacts on victims, after their voluntary participation in VOM. These psychological impacts can be operationalized as any changes or emerging psycho-emotional aspects, which differ from the previous state of victimization caused by the crime (i.e., prior to RJ intervention). Thus, the analysis of the selected studies focused on psychological effects of VOM such as: (a) post-traumatic stress symptoms (PTSS) (Lloyd & Borril, 2019), (b) negative emotions/emotional states (e.g., anxiety, anger, sadness, fear, guilt, distress) (Strang, 2002), (c) aspects created by victimization process (e.g., insecurity, lack of control, injustice, lack of self-esteem) (Gustafson, 2005; Kellas & Manusov, 2003), (d) negative perceptions about oneself and its offenders (Umbreit et al., 1994), and (e) emotional needs resulted from the crime (i.e., (1) need of information to better understand what happened and why, (2) need to be more involved in the justice process, (3) need to express their emotions and to be validated, (4) need of being empowered and to achieve emotional overcome of the situation) (Strang, 2002).

Regarding the included publications, the studies considered in this systematic review were restricted to those published in the period between 2000 and 2020, in academic and scientific journals with peer-review, as well as in specialist journals, excluding theoretical, commentary or opinion articles and gray literature, which includes conference proceedings, conference papers, unpublished works and masters’ and doctoral dissertations. Excluding gray literature was a practical decision, related to the difficulty of having a comprehensive database with this kind of literature, which could compromise reproducibility.

### Information Sources

The systematic search for relevant literature was carried out through the following electronic databases: Web of Science, Scopus, PubMed, and EBSCOhost. In resource aggregator portals were selected and activated databases as: Criminal Justice Abstracts, PsycTherapy, PsycINFO, PsycARTICLES, Psychology and Behavioral Sciences Collection, PEP Archive, Academic Search Complete, ERIC and Medline. The search of the present work was conducted in February 2021 and was limited to articles written in English, Spanish, and Portuguese languages.

### Search Strategy for the Identification of Relevant Articles

The present review sought to capture the studies conducted on victims’ outcomes in RJ processes, but contrary to previous reviews, these results did not refer merely to satisfaction levels. Instead, the results focused on the range of psychological effects. In this sense, the search terms were developed based on the structure discussed above, linking RJ practices to victims, who voluntarily participated in VOM based on RJ theories, and from where the effects of a psycho-emotional nature arising from these processes are evidenced. Thus, the following search equation was created: (*restorative justice***or***restorative practice***or***restorative approach***or***victim–offender conference***or***victim–offender mediation*) **AND** (victim*)**AND** (outcome*orbenefit*oreffect*orimpact*orconsequence*). The articles determined by this search equation were scrutinized by their title and abstract to include only relevant studies that met the aforementioned inclusion criteria. From the studies selected for analysis, their bibliographic references were also manually examined, to explore, if pertinent, other possibilities for inclusion (by backward citation search method) (Garrard, 2011).

### Screening and selection process

After obtaining all the references identified by searching databases, the sequential process of selecting the articles to be included in this systematic review began. Thus, to consistently proceed methodically, the PRISMA Statement tool was used. The instrument consists of 27-items checklist and includes a flow diagram, which facilitates the synthesis and presentation of the entire data management process of conducting systematic reviews (Liberati et al., 2009). Thus, following the flow diagram tool, we performed four steps:

(1) *identification* of the articles, referring to the total number of articles retained from electronic databases search, the total number found in the manual search (i.e., backward citation search) and the final number after removing the duplicates;
(2) *screening* articles, accomplished by reading its titles and abstracts;
(3) assessment of *eligibility* of the articles, performed by its full-text reading to select those that should be included or excluded, based on the criteria previously defined;
(4) *inclusion* of relevant articles, presenting the final number of studies included for the qualitative analysis.

To reduce observer bias, the search and selection of articles for analysis were carried out by two independent researchers. Finally, the divergences were discussed until there was a consensus.

### Quality Assessment

After identifying the eligible studies to be included in this systematic review, their empirical quality (EQ) was assessed, to better control possible biases in the evaluations and avoid erroneous extrapolations of the results. To this end, the Mixed-Methods Appraisal Tool (version 2018)—MMAT (Hong et al., 2018) was used. This tool establishes two initial criteria, which aim to validate its application to the study included, and five criteria that aim to assess its EQ. The choice of this tool was due to these five criteria that vary in a specific way depending on the type of study in question, enabling the assessment of the quality of studies with different methodologies (qualitative, quantitative, or mixed-method). Regarding quantitative methodology studies, the MMAT analysis is also sensitive to several designs, enabling the differentiation between randomized controlled trials, non-randomized and descriptive.

For practical purposes, we assumed that all the five criteria contribute equally to the analysis of the EQ of the studies. Therefore, to quantify the quality of each study, one point was attributed for each of the five quality criteria met.

Like the previous steps, to overcome possible confirmatory biases, the evaluation of the articles’ EQ was carried out by two independent researchers, and the divergences were discussed until there was an inter-judge agreement.

### Data Collection and Analysis

Finally, we extracted and analyzed the content of the data, documenting and exploring the characteristics of the studies identified in categories of interest, namely: (a) identification of authors and year of publication; (b) study goals, (c) type and design of research, (d) origin and composition of the sample; (e) type of intervention; (f) psychological outcome of interest involved; (g) and main results referring to the previously identified psychological outcome, which allows to answer to the research question of this work.

## Results

### Included studies

**Figure 1.**
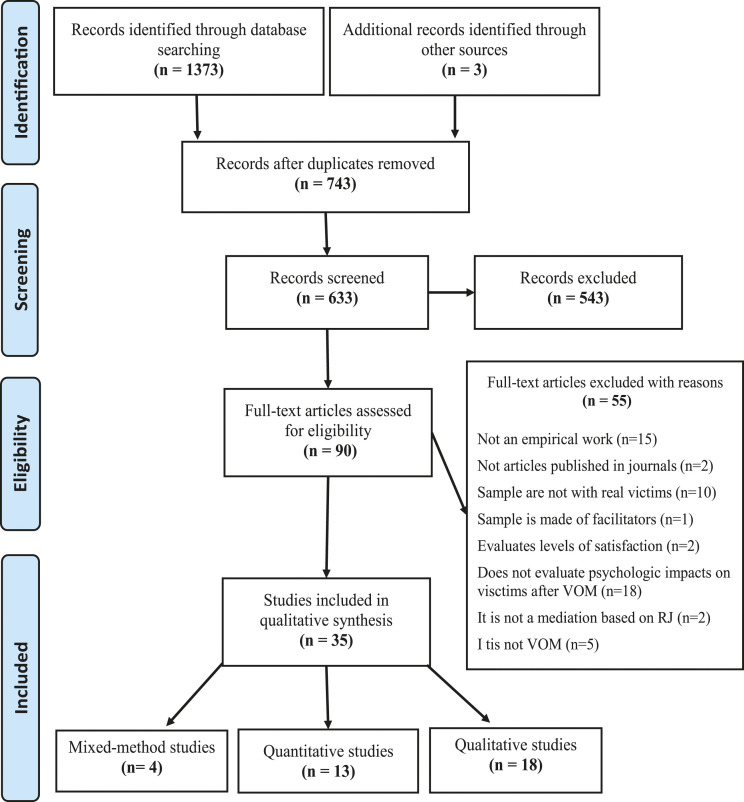
Flowchart outlining literature review search and selection process (adapted from Liberati et al., 2009).

From the initial search, 1373 articles were obtained, which were reduced to 630 after removed duplicates. A further three empirical studies were identified (Beck et al., 2015; Tamarit & Luque, 2016; Wemmers & Cyr, 2005J. A. Wemmers & Cyr, 2005) following the reading of two previous systematic reviews (Choi et al., 2012; Lloyd & Borril, 2019) and the bibliographic analysis of articles relevant to this review (i.e., by backward citation search method) (Avieli et al., 2021). Thus, a total of 633 studies were screened based on their title and abstract, resulting in 90 studies identified by the eligibility analysis through a complete reading of the articles. From this set of articles, 55 were excluded for not respecting the inclusion criteria stipulated for this review, namely: a) 15 articles for not being an empirical work; b) two references because they are not articles published in peer-reviewed scientific or academic journals; c) 10 articles because it is not a sample composed by real victims of crimes; d) one article because it is from the perspective of the facilitators and not the victims; e) two articles for merely assessing the satisfaction level of victims; f) 18 articles for not referring to psychological impacts on victims after their participation in restorative VOM; g) two articles for addressing mediations that are not based on RJ theories and h) five articles for not specifically focusing on the restorative practice of VOM. At the end of the search process, screening and selection of eligible studies, a total of 35 studies were included in this systematic review to carry out a content analysis of the results (Figure 1).

As for the assessment of the articles EQ, it was possible to verify that all of them respect the criteria for the MMAT application (i.e. all articles clearly established their research questions and sought to adopt appropriate methodologies to obtain relevant data). Most of the studies included have a satisfactory EQ, fully or almost fully meeting the MMAT criteria. However, five of these studies only meet one or two of the five stipulated quality criteria, scoring lower (Davis, 2009; Hargovan, 2010; Poulson & Elton, 2002; Tamarit & Luque, 2016; Wemmers & Cyr, 2005J. A. Wemmers & Cyr, 2005). The methodological weaknesses (or omissions) were related to the sample representativeness, sampling strategy, contemplation of parasite variables and/or the lack of complete results. Despite this, we decided not to exclude these articles by their lower quality, not only because it could lead to a form of selection bias (e.g., collider-stratification bias), which for some authors should be avoided (e.g., Stone et al., 2019), but also because the results of these articles are in line with the rest of the studies included.

### Characteristics of the included studies

In this systematic review, 35 studies were included; 13 are quantitative studies, 18 are qualitative, and four are mixed-method studies (Table 1).

**Table 1.** Summary of studies included for review.

Study Id	Sample Origin	Main Goals of the Study	Participants (N; Type of Crime Suffered)	Data Collection	Psychological Outcome	Main Results	MMAT (EQ)
*Quantitative randomized controlled trials*							
Angel et al., 2014	UK	Examine the impact of face-to-face restorative justice conference between victims and their offenders on post-traumatic stress	*N* = 192 (89 RJ participants; 103 control) burglary or robbery (14% with physical damage, 15% with verbal threat)	The Revised impact of event Scale (IES-R) (Weiss & Marmar, 1997)	Post-traumatic stress symptoms (PTSS)	Significant reduction of PTSS (i.e., avoidance (fear) (*p* = .024; *d* = .335); intrusion (distress) (*p* = .04; *d* = 4.447)No significant differences in reactivity (anxiety) (*p* > .05)	4
Gal & Moyal, 2011	Australia	Evaluate to what extent RJ is more satisfying than courts and compare between young victims and adult victims	*N* = 232 (196 adults; 36 young) Drink-driving, shoplifting, property crimes and violent crimes (86% young e 25% adults)	Unspecified Scale (Likert type)	Emotions (anger, sadness, frustration)	For all victims, there were significant effects of the restorative intervention in relation to the process (more positive emotions after RJ) (r (230) = −0.16, *p* = 0.017). This positive effect varies between the different age groups, tending to be (although not significant) smaller than the size of the effect in the younger victim group (*d* = −0.28, *p* > .05)	4
Sherman et al., 2005	Australia and UK	Analyze a RJ program (Jerry Lee program) for effect on victims	*N* = 146 (70 RJ; 76 control) property crimes and violent crimes	Questionnaire (Likert type)	Emotions (guilt, anger)	No significant difference in the sense of self-blame (*p* > .05). Less desire for revenge (anger) toward the offender (*p* < .001)	3
Sherman et al., 2015	Australia and UK	Assess the outcomes of the Jerry Lee program on victims and offenders in 12 randomized trials	*N* = 1179 (of which 400 control, that is, victims who participate in conventional justice) property crimes and violent crimes	Unspecified questionnaire from project RISE	Emotions (anger/anxiety) PTSS	Less anxiety (*p* < .001); less anger (about the crime) (*p* = .001). No significant difference in desire for revenge against the offender (*p* > .05) 49% less PTSS (effects prevail 10 years after RJ intervention)	3
*Quantitative non-randomized *							
Beven et al., 2005	Australia	Evaluate the ability of a restorative justice process to adequately attain process outcomes associated with restorative justice goals	*N* = 83 (36 RJ; 47 control) theft, assault, aggression, fraud, and disorderly conduct	2 items—Likert (perception of security); n-items (unspecified)—Likert (sense of control); 1 item—Likert (feelings about the offender)	Emotional needs (information/sense of security/sense of control) perception about the offender	Higher perception of security(t (50) = 4.57; *p* < .001) Better understanding of what happened and the legal process itself—sense of control (t (54) = 8.12; *p* < .001) more positive feelings about the offender (t (55) = 9.53; *p* < .001)	3
Boriboonthana & Sangbuangamlum, 2013	Thailand	Analyze the effects of restorative justice on victims and offenders	*N* = 479 (278 RJ; 201 control) trafficking, property, crimes against body, against life, sexual-related	Questionnaire (unspecified) and coded interviews	Sense of justice; emotional needs (expression/information; (*why me?*)); perception about the offender emotions (fear)	Higher sense of justice (93.8%); greater satisfaction with the process taking into account responses to emotional needs (92%); more positive perception of the offender (63.3%) less fear of the offender (11.5%)	3
Calhoun & Pelech, 2013	Canada	Compare the impact on victims of restorative and conventional approaches to juvenile justice	*N* = 67 (14 RJ; 53 control) property, violent crimes or both	8 items—Likert (damage repair) 6 items—Likert (offender responsibility) 7 items—Likert (hope in the future)	Emotional distress; emotions (guilt); closure	Less emotional distress (t (64) = 2.53, *p* < .05) reduction of the guilt of oneself or the offender (t (64) = 2.60, *p* < .05) less negative thoughts, aiming the sense of closure, involving the feeling of relief and the idea of being able to go on (t (64) = 2.02, *p* < .05)	4
Davis, 2009	USA	Examine how restorative practices affect victims and offenders	*N* = 465 (259 RJ; 206 control) assault (51%), burglary (29%), grand larceny (7%) others (13%)	Coded interviews	Emotions (anger, fear); emotional needs (expression) sense of justice	Less anger felt (χ2 = 117.65, *p* < .001) less fear felt (χ2 =12.30, *p* < .001) 65% of victims reported that felt heard (*d* = 22.09, *p* < .001) 76% reported more sense of justice (*d* =11.16, *p* < .001)	2
Koss, 2014	USA	Evaluate the impact of RJ program—RESTORE on sexual violence victims	*N* = 22 sexual violence	Questionnaire 17-item—Likert (PTSS) (Foa et al., 1993) Questionnaire unspecified—Likert (RJ experience)	PTSS (emotional distress and arousal) emotional needs (validation)	Significant decrease of PTSS between pre-conference (82%) and post-conference (66%) (*p* < .05) emotional distress (M = 7.17 pre-conference, and M = 6.50, post-conference; t = 2.06, *p* > .05) Arousal (M = 9.22 pre-conference e M = 8.22, post-conference; t = 1.98, *p* > .05) the results pointed to the importance of validation of the suffering in these victims	3
Poulson & Elton, 2002	USA	Analyze the levels of satisfaction and other attitudes among participants of RJ program of Utah, understanding how attitudes of victims and offenders differ from each other	*N* = 170 Unespecified crimes	Questionnaires unspecified—Likert and dichotomic type	Emotional needs (expression/validation/information); closure; perception about the offender	95% of victims felt heard; 94% pointed out the opportunity to express their own feelings; 83% achieve answers for what have happened; 91% achieve a sense of closure; 68% made a more positive opinion about their offender	1
Strang et al., 2006	Australia and UK	Analyze responses to retrospective interviews of victims participating in restorative conferences, in order to assess to what extent these practices helped to alleviate their psychological damage	*N* = 210 property, violent crime, burglary, theft, robbery, and assault	Retrospective interviews (coded)	Emotions (fear, anger) emotional needs (expression/information (*why me*?)) perception about the offender	Decreased of the fear felt (>50%) and anger after the RJ meeting (>50%). More empathy felt about the offender (>50%). The majority of victims referred that RJ process meet their information needs (>90%) and facilitate their expression of emotions (>90%)	3
*Quantitative descriptive*							
Hargovan, 2010	South Africa	To outline the findings of a pilot study on how victims of domestic violence experience restorative justice processes (victim–offender mediation)	*N* = 205 domestic violence (188 women +17 men)	Coded interviews (Kraska & Neuman, 2008)	Emotions (fear); emotional needs (expression/validation); closure	187 victims described their participation on RJ as good or very good (classification was based on). Greater sense of security and less fear; opportunity to express feelings and felt heard sense of overcome (closure) 187 participants considered their interaction with the offender more positive (good or very good) after the RJ meeting	2
Tamarit & Luque, 2016	Spain	Assess the degree to which a mediation program is able to achieve the objectives of restorative justice from the point of view of victims’ needs	*N* = 121 crimes against family, against property, against freedom, violent offences, others	Questionnaire unspecified	Emotional needs (validation/expression/involvement). Emotions (anger, fear, sadness, impotence and lack of control) empowerment	Victims valorized: (1) the possibility of being heard, (2) the opportunity to explain the impacts of the crime to their offender, (3) the chance to be more involved in their own process. Every negative emotion (specially anger and impotence) decreased after RJ mediation (*p* < .001), decreasing the overall emotional distress and increasing the sense of empowerment on victims	1
*Qualitative studies*							
Armstrong, 2012	UK	Explore victims’ perceptions about restorative justice processes	*N* = 35 assault and other crimes with low severity	Semi-structured interviews	Emotional needs (expression/information (*Why me*?)) perception about the offender	Opportunity to get answers for their victimization; greater sense of being heard; more positive perception about their offender after the RJ meeting	5
Beck & Kropf, 2015	USA	Explore ways that RJ can be used to help older adults bring closure to past wounds and harms, and to heal from trauma experiences	*N* = 2 homicide	Direct observation	PTSS closure	The observations result on a reduction of PTSS; mention to the importance of participating in a RJ meeting form emotional overcome (closure)	4
Bolitho, 2015	Australia	Describe RJ practice through the lens of victims who participated in a victim–offender conference and explore its impacts	*N* = 19 murder, manslaughter, driving offences leading to death, and sexual offences	Analysis of the departmental case files with information about victim–offender mediation; in-depth semi-structured interviews	Emotional needs (security/information/expression/validation); empowerment; closure	95% of the victims felt that RJ processes meet their needs (sense of security, information, expression and validation); victims reported a sense of control (empowerment) and emotional overcome (closure); the intervention effects prevailed after 5 years	5
Bolitho, 2017	Australia	Examine how restorative justice works to alleviate the emotional effects of crime on victims	*N* = 20 Serious crimes (assault, murder, sexual abuse, manslaughter)	In-depth interviews	Emotions (anger, fear, sadness)	Participation in RJ led to a great decrease (complete elimination) of some negative emotions (i.e., anger, fear and sadness), resulting in higher emotional well-being	5
Coates et al., 2006	USA	Assess the impact of RJ dialogue in seven case studies	*N* = 7 hate crimes	Direct observation	Emotions and beliefs	RJ dialogue offers at the very least an opportunity for replacing hate with understanding and respect	5
Halsey et al., 2015	Australia	Examine the key processes and outcomes of a pilot RJ mediation project	*N* = 10 common assault, indecent assault, criminal damage, illegal use of a motor vehicle, and break and enter	Observation + semi-structured interviews	Emotional needs (validation/expression/information (why me?)); closure	Victims’ report having satisfied their emotional needs, namely, their need to express themselves and be heard (validated) and to get answers about their victimization (why me), contributing to the feeling of emotional overcome (closure)	5
Jacobson et al., 2012	Sweden	Understand which benefits victims can achieve when they communicate with offenders in the framework of restorative justice (how victims interact, communicate and position themselves in relation to the offender in mediation)	*N* = 26 robbery, assault, aggression, vandalism fraud	Direct observation (verbal interactions analysis based on Have, 1999 and Norrby, 2004)	Self-esteem; emotional needs (information); perception about the offender	Victims report the possibility of rebuilding a more positive self-esteem; victims report the importance of the encounter to better understand what happened to them; most victims appeared to show an empathetic interest in the perpetrator’s life; the benefit of mediation meetings was limited by attitudes of moral dominance	5
Lavin & Carrol, 2014Lavin & Carroll, 2014	Ireland	To describe an RJ response to an episode of offending in a small village involving multiple victims	*N* = 6 property crimes	Direct observation	Emotional needs (validation) empowerment	5 of the 6 victims reported having satisfied the need to be heard (to be validated); greater sense of empowerment in victims	5
Miller & Iovanni, 2013	USA	Explore the effectiveness of RJ’s dialogues between victim and offender occurring post-conviction	*N* = 1 domestic violence	Open-ended interviews	Emotions (guilt) empowerment	Victim reports better sense of control and empowerment; victim reports becoming aware that the crime was not her fault and that the aggressor is solely and directly responsible	5
Murhula & Tolla, 2020	South Africa	Investigate the effectiveness of RJ practices (i.e., victim–offender mediation) on victims of crimes	*N* = 5 violent crimes (attempted murder, armed robbery and sexual offence) and minor crimes (theft and fraud)	*Questerview* (based on Davies, 2009)	Emotional needs (information (why me?)/expression); closure	Victims reported the opportunity of express their emotions and get answers for their victimization; victims reported achieving a sense of emotional overcome (closure)	5
Tapp et al., 2020	UK	Describe the process and outcomes of RJ mediation between a detained patient with autism and a person he harmed	*N* = 1 Attempted murder	In-depth interview	PTSS	The victim reported that restorative encounter facilitated the management of the traumatic process and feeling less anxiety and a sense of relief at the end of the conference	5
Umbreit et al., 2000	USA	Report consequences of participating in RJ, more specifically in victim–offender mediation	Study 1: *N* = 280 RJ (+210 no RJ); study 2: *N* = 183 RJ (+140 no RJ); study 3: *N* = 34 RJ (+26 no RJ); property crimes (burglary)	Structured interviews	Sense of justice emotions (fear)	High levels of sense of justice were expressed by the victims participating in mediation; face-to-face mediation reduced fear of being victimized by the same offender	5
Umbreit et al., 2010	USA	Examine restorative meetings in Texas and Ohio (dialogue between victims of serious and violent crime and their offenders) and explore the experiences and impacts of these participants	*N* = 40 Serious and violent crimes (Felony assault, manslaughter, murder or attempted, sexual assault, theft, burglary, homicide, vehicular homicide)	Semi-structured interviews	Emotional needs (information/expression); emotions (fear e anger); perception about the offender; closure	31% reported getting answers for what happened; 31% reported a reduction on their negative emotions (fear and anger) 52% gotten a more positive perception about the offender 74% reported the RJ process helped with the emotional overcome (closure)	5
Umbreit &Vos, 2000	USA	Examine the experience of mediation session in capital murder case involving surviving family members	*N* = 3 homicide	In-depth interviews	Emotions (anger, fear); closure	Victims reported that after the restorative encounter they felt less anger and fear, and gotten a sense of emotional overcome (closure)	5
Umbreit et al., 2006	USA	Explore two programs of RJ, along with outcome data related to the experience of both crime victims and their offenders in mediated dialogue in the context of severely violent crime	*N* = 20 homicide, attempt murder, sexual assault, and theft/burglary	Semi-structured interviews	Emotional needs (information); emotions (anger); closure	Victims gotten answers about their victimization victims referred that during the RJ meeting they had the willingness to abandon hatred and put anger aside, and that process was because they felt sincerely remorse by the offender. Victims achieved a sense of emotional overcome after the meeting	4
Van Camp & Wemmers, 2013	Canada and Belgium	Explore the factors that contribute to victim satisfaction with RJ	*N* = 26 violent crimes (homicide, murder, manslaughter, bodily harm, aggravated assault, kidnapping, stalking and robbery)	Semi-structured interviews	Emotional needs (expression/validation/information, security); sense of justice; empowerment; closure	Victims had a greater sense of security, the possibility to express their emotions, a bigger sense of justice (validation) and gotten answers about their victimization; victims reported higher sense of control (i.e., empowerment) victims achieved an emotional overcome state	5
Walters, 2015	UK	Examine the therapeutic benefits that RJ meetings can engender for stakeholders of homicide	*N* = 1 homicide	In-depth interviews	Emotional needs (information) closure perception about the offender	Victim referred an emotional well-being through getting answers about the crime; victim reported RJ meeting important for grieving process and overcome (closure). A more positive perception about the offender after RJ meeting was achieved by the victim	5
Wemmers & Cyr, 2005J. A. Wemmers & Cyr, 2005	Canada	Examine the relationship between participation in victim–offender mediation and victim recovery	*N* = 59 Aggression (46%), assault (5%), threats (3%), robbery (20%), carjacking (12%), vandalism (10%)	Semi-structured interviews	Emotions (fear); sense of justice	All victims except one reported a reduction in their level of fear; 64.1% of victims referred a good sense that justice was done (validation), contributing to their psychological well-being	2
*Mixed-method studies*							
Barr, 2013	UK	To examine the impact of restorative initiatives on perceptions of legitimacy on victims	*N* = 26 Unspecified crimes	Questionnaire 50 items—Likert (Gaboury & Sedelmaier, 2007); semi-structured interviews; direct observation	Emotional needs (expression/validation) sense of justice	Higher sense of validation (i.e., that was heard and could express their feelings) (τ = .40, *p* < .05) greater sense of justice (damage was recognized) (τ = .46, *p* < .05)	5
Bolívar, 2013	Spain and Belgium	Describe the findings of a mixed-method study carried out in the context of victim–offender (direct and indirect) mediation	*N* = 124; *N* = 50 qualitative (Spain) *N* = 74 quantitative (Belgium—39; Spain—35); qualitative sample: Physical assault, battery, robbery or attempted, threat, theft, attempted murder, other. Quantitative sample: Physical assaults, robbery or attempted thefts, battery, murder, arson, rape, threat and harassment, stalking	Open-ended interviews (based on Creswell & Plano, 2007) Questionnaires: 1) victim restoration Questionnaire—VRQ; 2) post-traumatic growth Inventory—PTGI; 3) social Acknowledgment Questionnaire—SAQ; 4) the self-blame scale—SB	Closure perception of support perception about the offender	Victims participating in direct mediation tended to score higher on all scales, suggesting that these participants had: Lower perception of damage (U = 384.5, *p* = 0.029), higher perception on social support (H = 6.298, *p* = 0.043). A more positive perception about the offender (U = 347.5, *p* > .05)	5
Helfgott et al., 2000	USA	Explore how RJ processes can be applied in a correctional setting	*N* = 15; homicide, rape, kidnapping, aggravated assault robbery and domestic violence	Questionnaire (unspecified Likert type); direct observation	Emotions; emotional needs (involvement)	Most victims felt more involved and comfortable (psychological well-being), considering the restorative experience as positive and emotionally transforming (t = −2.75, *p* = .02)	3
Pelikan, 2010	Austria	Examine the effectiveness of victim–offender mediation applied to cases of partnership violence	*N* = 162 (quantitative) + 21 (qualitative) Partnership/domestic violence	Questionnaire (unspecified); direct observation; interviews	Empowerment	The more prominent result on victims’ participants is the greater sense of empowerment 65% of the victims referred that they felt more self-confident and stronger	4

From these studies, it was possible to ascertain a total of 4697 participants, from 11 different countries, namely: South Africa (Hargovan, 2010; Murhula & Tolla, 2020); Australia (Beven et al., 2005; Bolitho, 2015, 2017; Gal & Moyal, 2011; Halsey et al., 2015; Sherman et al., 2005, 2015); Austria (Pelikan, 2010); Belgium (Bolívar, 2013; Van Camp & Wemmers, 2013); Canada (Calhoun & Pelech, 2013; Van Camp & Wemmers, 2013; Wemmers & Cyr, 2005J. A. Wemmers & Cyr, 2005; 2006); Spain (Bolívar, 2013; Tamarit & Luque, 2016); USA (Umbreit et al., 2000, 2006, 2000; Beck et al., 2015; Coates et al., 2006; Davis, 2009; Helfgott et al., 2000; Koss, 2014; Miller & Iovanni, 2013; Poulson & Elton, 2002; Umbreit & Vos, 2000); Ireland (Lavin & Carroll, 2014); United Kingdom (Angel et al., 2014; Armstrong, 2012; Barr, 2013; Sherman et al., 2005, 2015; Tapp et al., 2020; Walters, 2015); Sweden (Jacobson et al., 2012); and Thailand (Boriboonthana & Sangbuangamlum, 2013).

In addition, as defined, all participants covered by the studies included in this work were real victims of crimes, either of low severity (i.e., property crime, arson, disorderly conduct, trafficking, theft, burglary, fraud) or of high severity (i.e., assault, assault/offense to bodily harm, murder or involuntary manslaughter, sexual crime, domestic violence, hate crimes). As a result of these crimes, 3611 of these victims voluntarily participated in VOM, based on the general principles and values of RJ. The correspondence between the participants of the different studies and the crimes of which they were victims is summarized in Table 1.

Regarding the demographic features of the participating victims, although the results in general do not show significant differences between gender and psychological impacts after the restorative meetings, some of the studies chose to use a sample mostly or even exclusively constituted by female victims (Hargovan, 2010; Koss, 2014; Miller & Iovanni, 2013; Pelikan, 2010; Van Camp & Wemmers, 2013). Concerning age, although the vast majority of the included studies analyzed victims in adulthood, two of them also examined juvenile victims (i.e., under the age of 18) (Calhoun & Pelech, 2013; Gal & Moyal, 2011). One of these studies founded some differences (although not statistically significant) in the positive impacts of RJ between different age groups, with a tendency to smaller effect in the group of younger victims (*d* = −0.28, *p* > .05) (Gal & Moyal, 2011).

### Main psychological impacts on victims

The main results of the included studies showed that, regardless of their design, the psychological impacts identified focused on the same aspects (Table 2). Thus, it is possible to ascertain the consistent and significant decrease in post-traumatic stress symptoms after victims’ participation in VOM (Angel et al., 2014; Beck et al., 2015; Koss, 2014; Sherman et al., 2015; Tapp et al., 2020). Angel et al. (2014) found that, except for levels of anxiety (*p* > .05), this decrease was more expressive in levels of distress (*p* = .04; *d* = 4.447) and fear (*p* = .024; *d* = .335) in victims who undergo restorative processes (i.e., VOM), when they compared with victims that go through conventional legal processes and that suffered the same type of crime.

**Table 2.** Summary of extracted data and critical findings of the main psychological impacts of RJ on victims.

Extracted Data	Description	N
Sample size	Minimum	1
	Maximum	1179
		**N of studies**
Study design	Quantitative (randomized controlled trials)	4
	Quantitative (non-randomized)	7
	Quantitative descriptive	2
	Qualitative	18
	Mixed-methods	4
Main psychological impacts	Decrease of PTSS	5
	Decrease of negative emotions (fear/anger/guilt/anxiety/distress)	17
	More positive perceptions about the offender	9
	Satisfaction of emotional needs (information/expression/validation)	21
	Achievement of emotional overcome (*closure*)	12
	Recovery from aspects related with victimization process	8

Many of the results also point out considerable reductions in negative emotions expressed by victims (fear, anger, guilt, anxiety, distress) after their participation in RJ mediations/conferences (Tables 1 and 2). The results consistently showed that the negative emotion of anger felt by victims toward their offenders is lower after VOM, compared to the levels of anger felt by victims who were subjected to conventional justice interventions. In Davis (2009) study, this aspect showed to be significant (*p* < 0.01; *d* = 117.65). Furthermore, this evidence has shown to be persistent over the years (Sherman et al., 2015). Alongside this, there is a decrease in the desire for revenge by the victims-participants (Sherman et al., 2005, 2015), highlighting changes in the perception they have regarding their offenders after VOM and assuming more positive attitudes (Armstrong, 2012; Beven et al., 2005; Boriboonthana & Sangbuangamlum, 2013; Jacobson et al., 2012; Poulson & Elton, 2002; Strang et al., 2006; Umbreit et al., 2010; Walters, 2015). The results also reveal that victims who participated in direct restorative mediations show a more positive perception about the offender, compared to victims who refused to participate, or who participated in indirect mediations (Bolívar, 2013).

As for the recovery of aspects created by the victimization process, some studies show that the participation of victims in VOM promotes a reduction in feelings of helplessness about of what has happened (Miller & Iovanni, 2013; Tamarit & Luque, 2016; Van Camp & Wemmers, 2013), along with an increased perception of security (t (50) = 4.57; *p* <.001) (Beven et al., 2005) and self-esteem (Jacobson et al., 2012). Besides, studies reported the development, during the restorative process, of a renewed sense of control and empowerment (Bolitho, 2015; Lavin & Carrol, 2014Lavin & Carroll, 2014; Pelikan, 2010).

The results show that VOM practices meet the multiple emotional needs of victims, reporting a greater involvement in the justice processes (Helfgott et al., 2000) and referencing the satisfaction of the need for information (Jacobson et al., 2012; Umbreit et al., 2006), expression, and validation (Armstrong, 2012; Barr, 2013; Beven et al., 2005; Bolitho, 2015; Boriboonthana & Sangbuangamlum, 2013; Halsey et al., 2015; Hargovan, 2010; Koss, 2014; Lavin & Carrol, 2014; Murhula & Tolla, 2020; Umbreit et al. al., 2000, 2010; Poulson & Elton, 2002; Strang et al., 2006; Tamarit & Luque, 2016; Van Camp & Wemmers, 2013; Walters, 2015; Wemmers & Cyr, 2005J. A. Wemmers & Cyr, 2005).

There are still many results in which victims report that participation in VOM gave them a sense of emotional overcome (closure), based on relief and emotional conditions to continue their lives (Beck et al., 2015; Bolívar, 2013; Calhoun & Pelech, 2013; Halsey et al., 2015; Hargovan, 2010; Murhula & Tolla, 2020; Poulson & Elton, 2002; Umbreit et al., 2006, 2010, 2006; Umbreit & Vos, 2000; Van Camp & Wemmers, 2013). This sense of closure has been shown to be a prevalent result over time (Bolitho, 2015).

## Discussion

The present work aimed to carry out a systematic evaluation of quantitative, qualitative, and mixed-method empirical production, to aggregate and examine dispersed information regarding the psychological impacts on victims who participate in restorative victim–offender meetings (VOM), namely, in mediations or conferences.

It was possible to verify that regardless of the study design (quantitative, qualitative, and mixed-method), there was an agreement regarding the psychological impacts identified on the victims participating in VOM. Thus, several studies report that victims who contacted their offenders in restorative meetings significantly exhibited a decrease in their post-traumatic stress symptomatology (PTSS) (Angel et al., 2014; Beck et al., 2015; Koss, 2014; Sherman et al., 2015; Tapp et al., 2020), which according to Lloyd and Borril (2019) is related to high levels of anxiety, distress and fear. The results of the included studies have consistently shown that after VOM there is a decreasing of the aforementioned levels on victims (Umbreit et al., 2000, 2006, 2010, 2006; Bolitho, 2017; Boriboonthana & Sangbuangamlum, 2013; Calhoun & Pelech, 2013; Coates et al., 2006; Hargovan, 2010; Helfgott et al., 2000; Miller & Iovanni, 2013; Strang et al., 2006; Tamarit & Luque, 2016; Umbreit & Vos, 2000; Wemmer & Cyr, 2005).

This evidence is in line with the victimology literature, which advocates that the narration of trauma, as happens in RJ practices, favors the reparation of victims, relieving their fears and anxieties (Neimeyer et al., 2006). In addition, the study by Angel et al. (2014) even shows that the decrease of PTSS, associated with levels of anxiety and fear, is more expressive in victims who undergo restorative practices, compared to victims who go through conventional legal processes. This fact is eventually based on the premise that restorative meetings represent an opportunity for the victims to expose themselves to direct contact with their offender(s) in a safe and controlled environment (EFRJ, 2020). Thus, if cognitive-behavioral psychological theories are evoked here, as Sherman et al. (2005) did, it can be said that this safe and controlled exposure to the traumatic stimulus (offender) can explain the decreases of fear levels felt by victims after VOM.

However, the results of the present study reveal divergences regarding the levels of anxiety in victims, after different types of intervention (restorative justice and conventional justice) (Angel et al., 2014; Sherman et al., 2015). This incongruity may refer not only to the fact that rates of hyperreactivity (associated with anxiety) consistently decrease over time after a traumatic event (Orth et al., 2006), but also to methodological issues related to the time intervals that are far between the occurrence of crimes and interventions (RJ or CJ), or between participation and data collection. This inference results from the observation that there was no temporal uniformity between the study of Sherman et al. (2015) and the study of Angel et al. (2014), with the latter having a longer period between the intervention and the data collection. In addition, none of these studies clarify the time interval between the time before the occurrence of crimes and the intervention. Thus, the importance of establishing the variable time in this type of study is highlighted, since shorter intervals between crime and mediation, and between mediation and data collection, can be more appropriate to observe the effects of interventions, especially with regard to levels of anxiety.

The results of the various studies included here also showed that the participant-victims expressed less anger toward their offenders after the restorative meetings (Bolitho, 2017; Davis, 2009; Gal & Moyal, 2011; Strang et al., 2003, 2006; Tamarit & Luque, 2016; Umbreit et al., 2006, 2010, 2006; Umbreit & Vos, 2000), thus reducing their desire for revenge toward to them (Sherman et al., 2005, 2015). In this way, we can think about the relevance of achieving one of the goals of VOM, which is the dialogue seeking compensation for the harm suffered by the victim, through the expression of remorse and apology by the offender (EFRJ, 2020; Zher, 2005). Besides, regarding the importance of the victims’ perceptions about the offenders' apologies (Choi et al., 2012), it is possible to state that the generality of the results of this study suggests that the demonstrations of offenders’ remorse were perceived by the victims as being authentic. This perception motivates forgiveness and psychologically impacts the victim in a positive and significant way, with this effect prevailing over time (Sherman et al., 2015). It is reasonable to say that reducing the emotion of anger and feelings of revenge toward the offender depends on the victims’ perception of sincere offenders’ remorse. Thus, it is important to highlight the relevance of safeguarding procedural issues such as offenders’ accountability for the crime committed, along with their suitability to participate in these restorative meetings.

Also, regarding the emotion of anger felt by victims, it was possible to identify evidence in Gal and Moyal (2011) study, that points to the possibility of population differences between adult and juvenile victims, with the latter group having expressed a less marked decrease in this emotion after the VOM (although this is not shown statistically significant by these authors). Such evidence seems to be based not only on differences in the operationalization of the RJ, which in the case of young victims necessarily involves the presence of family/guardians (Miers, 2003), but also on issues related to the conduct of mediation itself. In the study in question (Gal & Moyal, 2011), some of these young victims reported the dominance of their parents in mediation, diminishing their role in the conference. This imbalance caused in the sphere of power of young victims goes against the RJ theoretical basis and can compromise the effectiveness of these practices (EFRJ, 2020; Zehr, 2015). Still following this question, the study by Jacobson et al. (2012), pointed to the existence of limitations in the benefits obtained from restorative mediations, due to the morally dominant position taken by some of its victim-participants. In this sense, it is relevant to highlight what was mentioned by Choi et al. (2012), about the importance of a solid training of mediators, to maintain high quality standards of these professionals in conducting restorative meetings.

Besides, the emotional transformation regarding the victims’ guilt is highlighted, with results that point out to a decrease of self-blame related to the traumatic event experienced, after their participation in VOM (Calhoun & Pelech, 2013; Miller & Iovanni, 2013; Sherman et al., 2005). This evidence is congruent with what is considered by the RJ theory, which is based on a sincere and open dialogue about the offenders’ role in the crime, directly addressing their responsibility in it (EFRJ, 2020; Strang et al., 2006). Once again, it is worth emphasizing the need for procedural scrutiny supported by the assumption of responsibility for the crime and suitability that allows the offender to be a candidate for restorative practice.

Although the literature suggests that the self-blame felt by victims may vary depending on the type of crime suffered or the individual features of the victims and/or their offenders (Shapland & Hall, 2007), none of these variables were explored in the studies. However, although some of the studies included in this review used a majority, or even exclusively, female victims sample, this did not prove to be a relevant element of analysis for the purpose of this study, justifying this sample option with the specific demographic expression of certain crimes such as domestic violence or sexual violence (Hargovan, 2010; Koss, 2014; Miller & Iovanni, 2013; Pelikan, 2010; Van Camp & Wemmers, 2013).

Thus, the absence of analysis of this type of variables (i.e., type of crime and participants features) constitutes a methodological limitation, which raises the need for additional empirical production that explores the conditions in which the RJ can operate and become more psychologically beneficial to the participating victims. In the same way, it has to be noted that none of the included studies, from 11 countries and five different continents (with different cultures and historical backgrounds) examined diversity aspects of the studied populations (e.g., racial/ethnic characteristics and/or sociocultural contexts). This absence may have relevant implications in the interpretation of the outcomes of the studies, since recognizing that different groups may present differences between them is vital to developing research that not only reflects the needs of diverse populations, but also seeks to assess the impact of these tailored interventions (Bent-Goodley, 2021). Thus, not addressing diversity issues on the result analysis can be pointed as a limitation of each study included on this review (Tajima, 2021).

Nevertheless, the results also highlight the existence of changes in the perception that victims have about their offenders after VOM, taking on more positive and empathetic contours (Armstrong, 2012; Beven et al., 2005; Boriboonthana & Sangbuangamlum, 2013; Jacobson et al., 2012; Poulson & Elton, 2002; Strang et al., 2006; Umbreit et al., 2010; Walters, 2015). This fact is in line with what is defended by Braithwaite (2002), when he states that the wealth of restorative VOM lies in the humanization of the process and the consequent change of perception about the offender, which transforms the ‘criminal’ into a person who committed a crime and has its own context.

The results of this review showed, in a transversal way, that VOM in the RJ context meet the emotional needs of victims, resulting from their victimization processes. Thus, the participant-victims revealed to feel a greater involvement in their own processes (Helfgott et al., 2000), allowing them three key situations to overcome the victimization experience: (a) to obtain a better understanding of what happened (i.e., need for information) (Jacobson et al., 2012; Umbreit et al., 2006), (b) to have the opportunity to express their emotions and thoughts regarding the crime suffered (i.e., need for expression), and (c) to feel that they are heard and that their suffering is validated, contributing to a greater perception that justice has been done (i.e., need for validation) (Armstrong, 2012; Barr, 2013; Beven et al., 2005; Bolitho, 2015; Boriboonthana & Sangbuangamlum, 2013; Halsey et al., 2015; Hargovan, 2010; Koss, 2014; Lavin & Carrol, 2014Lavin & Carroll, 2014; Murhula & Tolla, 2020; Poulson & Elton, 2002; Strang et al., 2006; Tamarit & Luque, 2016; Umbreit et al., 2000, 2010; Van Camp & Wemmers, 2013; Walters, 2015; Wemmers & Cyr, 2005J. A. Wemmers & Cyr, 2005). Such results are consistent with the goal of a more flexible and humanized version of justice, centered on the people who participate in RJ meetings (Umbreit et al., 1994).

As a result, the data collected in this review also reveal that through VOM, victims not only achieve recovery from a feeling of impotence and/or lack of control over what has happened (Beven et al., 2005; Miller & Iovanni, 2013; Van Camp & Wemmers, 2013), but also re-establishing their perceptions of security (Beven et al., 2005) and self-esteem (Jacobson et al., 2012), as well as developing a sense of empowerment (Bolitho, 2015; Lavin & Carrol, 2014; Pelikan, 2010; Tamarit & Luque, 2016). Thus, as stated by Kellas and Manusov (2003), these results from the RJ are essential in the process of transformation from victim status to survivor status, which is imperative in the emotional recovery of a traumatic event (i.e., the crime).

In this sense, the results also highlight the evidence that participation in VOM provides victims with a sense of emotional overcome of what happened (closure), focusing on aspects such as relief and emotional strength to continue their lives (Beck & Kropf, 2015; Bolívar, 2013; Calhoun & Pelech, 2013; Halsey et al., 2015; Hargovan, 2010; Murhula & Tolla, 2020; Poulson & Elton, 2002; Umbreit et al., 2006, 2010, 2006; Umbreit & Vos, 2000; Van Camp & Wemmers, 2013). Interestingly, the results related to emotional overcome are consistently present in studies in which the participants were victims of crimes with a high degree of severity and violence (e.g., murder, physical, or sexual assault). In the work by Walters (2015), the case study participant even mentions that VOM was shown to be preponderant in her grief process. This fact gains consistency in the literature if we return to what was mentioned by Hoff and Hoff (2011), that the meaning of closure implies the acceptance of losses, which is a central component for the emotional overcome process of traumatic events, where there was little or no control (Hayes, 2004).

In addition, the study by Bolitho (2015) highlights that this sense of closure evidenced by victims participating in RJ conferences is not a transitory effect, but one that persists over time. In this way, it is possible to come up with the idea that RJ practices may consist of processes of deep and significant emotional change for the victims who participate in them.

In short, it can be said that, in general, the evidence found in this review is predominantly positive, focusing not only on the reduction of negative emotions of victims regarding their offender and crime, but also on strengthening them at an emotional level. These effects seem to be provided by the core element of restorative meetings of valuing victims and their needs resulted from victimization processes.

## Limitations and Future Perspectives

At a methodological level, it was possible to identify an important limitation in the development of this systematic review namely the option of not including gray literature, which might imply the possibility of relevant studies having been left out. In this sense, it is critical to mention the need to develop new reliable methodological tools that allow the reproducible inclusion of gray literature in future systematic reviews.

Regarding the conceptualization of this systematic review, although it supports the claim that RJ is an approach that seeks to meet the victim and their needs, benefiting them positively at a psychological level, some limitations can also be identified here (Table 3).

**Table 3.** Summary of limitations and implications of the review for practice, policy, and research.

Limitations of the Review	Implications for Practice and Future Research
• Gray literature (GL) was not included	• Develop new tools that allow the reproducible inclusion of GL in future systematic reviews
• The conditions for RJ application may be not always available	• Need previous check of procedural aspects for RJ eligibility such as the offenders’ accountability for the crime committed
• Included studies did not control some relevant variables	• Future research should focus and control important variables such as personal features of participants, mediator training, time intervals, crime severity, parallel psychological treatments
• Included studies did not examine diversity aspects of the studied populations	• Future research should address diversity aspects of the populations under study

In this work, it was possible to verify the importance of the previous procedural scrutiny for the RJ practice eligibility, namely the existence of accountability for the crime committed by the offender. However, when the conditions for the implementation and execution of VOM are met, the offender develops remorse and awareness, which according to Latimer et al. (2005) generally culminates in a reduction in recidivism rates criminal. As a result, beneficial and lasting psychological impacts for the victim are observed (Sherman et al., 2015). However, these effects seem to vary depending on the mediator, which may constitute a limitation to the analysis of the studies. Thus, it is imperative that future studies take into account the quality of the VOM and the professionals who mediate them, as this is something that can influence the effects of restorative interventions on victims who participate in them. In this sense, it is important to raise the issue of the need to standardize the training of mediators, as well as to assess their professional quality, in order to maximize the beneficial impacts for the parties involved in restorative practice.

In addition, it was also possible to identify some limitations related to the results obtained by the included studies. One of these limitations refers to the fact that the time variable was not considered on many of the studies included, that is, the time interval between the occurrence of crimes and the interventions (restorative or conventional), as well as between the VOM and the data collection. The divergences found in the results are based on this limitation.

Other variables that proved to be relevant for the analysis of the results of the included studies, but which none of them addresses directly, are the features of the participating victims and/or offenders and the severity of the crimes. If, on the one hand, the RJ participants features are relevant as they influence emotional issues (such as self-blame on the victims), on the other hand, so is the awareness of how the crime severity affects the impacts of RJ interventions, since the literature is contradictory regarding this aspect (Daly, 2005; Strang, 2002). In the same way, none of the included studies examined diversity aspects of the studied populations, which may have implications in the interpretation of their outcome results, as previously discussed. This constitutes a limitation of each individual study included in this review (and consequently a limitation of this review itself), unveiling a gap in the RJ literature, which should be addressed in future research (Tajima, 2021).

Furthermore, taking into account the needs and emotions emerging from the victimization processes, it is still worth pointing out as a limitation that no study considered whether the victim benefits from parallel psychological/psychiatric care. This may affect the way that VOM impacts psychologically these victims and may constitute itself as a confounding variable. In this sense, future studies should integrate the control of possible confounding variables, such as: (1) the time between the occurrence of the crime and the intervention, (2) the time between the VOM and the evaluation of its effect, (3) the existence of parallel psychological treatments, (4) the personal features of the participants (victims and offenders) and, (5) the crime severity. This additional scientific production becomes pertinent in the future, as it would improve empirical rigor and provide important clues about the resources and circumstances in which VOM operate and enhance psychological benefits for victims. In addition, RJ research can play an important role in prioritization and decision making regarding the RJ limited resources, as in to inform and sensitize public opinion about this kind of processes, in order to achieve greater involvement of the community (Costa, 2009).

Thus, this review has relevant implications for optimizing the implementation of restorative programs, since we aimed to provide a systematic listing of the main impacts and benefits of RJ on victims, this way contributing to the enrichment of the literature in this research area.

## Note

1 In this work the term *survivor* is based on the terminology referred by the SAKI Project (financed by the U.S. Department of Justice) and refers to a term of empowerment to convey a person who started a healing/recovery process after experiencing a crime.
